# Chronobiology and Chronotherapy in Inflammatory Joint Diseases

**DOI:** 10.3390/pharmaceutics13111832

**Published:** 2021-11-02

**Authors:** Francesco Ursini, Alfredo De Giorgi, Martina D’Onghia, Roberto De Giorgio, Fabio Fabbian, Roberto Manfredini

**Affiliations:** 1 Medicine & Rheumatology Unit, IRCCS Istituto Ortopedico Rizzoli, 40136 Bologna, Italy; francesco.ursini2@unibo.it (F.U.); martina.donghia@gmail.com (M.D.); 2 Clinica Medica Unit, Department of Medical Sciences, University of Ferrara, via L. Borsari 47, 44121 Ferrara, Italy; degiorgialfredo@libero.it; 3 Internal Medicine II Unit, Department of Translational Medicine, University of Ferrara, 44121 Ferrara, Italy; dgrrrt@unife.it

**Keywords:** chronobiology, inflammatory joint diseases, chronotherapy

## Abstract

Circadian rhythm perturbations can impact the evolution of different conditions, including autoimmune diseases. This narrative review summarizes the current understanding of circadian biology in inflammatory joint diseases and discusses the potential application of chronotherapy. Proinflammatory cytokines are key players in the development and progression of rheumatoid arthritis (RA), regulating cell survival/apoptosis, differentiation, and proliferation. The production and secretion of inflammatory cytokines show a dependence on the human day–night cycle, resulting in changing cytokine plasma levels over 24 h. Moreover, beyond the circadian rhythm of cytokine secretion, disturbances in timekeeping mechanisms have been proposed in RA. Taking into consideration chronotherapy concepts, modified-release (MR) prednisone tablets have been introduced to counteract the negative effects of night-time peaks of proinflammatory cytokines. Low-dose MR prednisone seems to be able to improve the course of RA, reduce morning stiffness and morning serum levels of IL-6, and induce significant clinical benefits. Additionally, methotrexate (MTX) chronotherapy has been reported to be associated with a significant improvement in RA activity score. Similar effects have been described for polymyalgia rheumatica and gout, although the available literature is still limited. Growing knowledge of chronobiology applied to inflammatory joint diseases could stimulate the development of new drug strategies to treat patients in accordance with biological rhythms and minimize side effects.

## 1. Introduction

Circadian rhythms are genetically encoded, near 24 h oscillations found in essentially every physiological process in the human brain and body [1] and reflect behavioral, metabolomic, proteomic, transcriptomic, acetylomic, and methylomic dynamics [2]. Circadian clocks are cell-autonomous, transcriptionally based, molecular mechanisms that enable organisms to prepare for changes in their physical environments and respond to environmental factors in a temporally appropriate manner [3]. The mammalian circadian timing system consists of three basic components: (i) input signals (environmental cues), (ii) a circadian oscillator as an intrinsic rhythm generator, and (iii) output rhythms. The suprachiasmatic nucleus (SCN), located in the anterior hypothalamus, consists of ap-proximately 20,000 neurons distributed across the SCN core and shell regions. The SCN receives environmental photic information by visual and nonvisual photoreceptors of the retina, reaches the SCN via the retinohypothalamic tract (RHT), and then synchronizes peripheral tissue clocks [4]. Such a self-sustaining nature requires the presence of a genetic mechanism known as the molecular circadian clockwork, and clock genes are required for generating and maintaining the circadian rhythm at either the organism or the cellular level. The first discovered clock genes were the Period gene (Per) in the fruit fly *Drosophila melanogaster* and the Clock gene in the mouse. In particular, Per encodes a protein that represses its own transcription, revealing a complex mechanism composed of activators that induce the expression of their own repressors, forming a negative feedback loop. This core loop consists of basic helix–loop–helix (bHLH) and Per-Arnt-Sim (PAS) heterodimeric transcriptional activators (CLOCK (circadian locomotor output cycles kaput) or its paralogue, NPAS2, with BMAL1 (brain and muscle Arnt-like protein 1)). Activators bind to E-box elements in the core clock repressors Period (Per1, Per2, or Per3) and Cryptochrome (Cry1 or Cry2) in mammals, which dimerize and then provide negative feedback to control their own transcription. This core loop is embedded and reinforced in other transcription feedback loops through CLOCK–BMAL1 activation of Rev-erbα and receptor-related orphan receptor (ROR) α [5].

As the internal regulatory elements of circadian rhythms, clock genes such as BMAL1, CLOCK, PER, CRY, nuclear receptor subfamily 1 group D member 1 (NR1D1), and retinoic acid ROR not only keep the clock oscillator ticking [6] but also participate in the regulation of physiological functions, including the sleep–wake cycle, heart rate, blood pressure, body temperature and, of crucial importance, hormone secretion and immune responses [7]. Regarding the latter, the 24 h rhythm has a profound impact on innate immunity, modulating the synthesis and release of cytokines/chemokines and the activity of the inflammasome [8,9], as well as on the adaptive immune system, influencing the differentiation and proliferation of both T and B cells [9]. Such daily rhythms are orchestrated not only by the principal circadian oscillator located in the SCN but also by other peripheral pacemakers located within organs, tissues, and cells, including the lungs, liver, kidneys, and leukocytes [10,11,12]. Coordination of this delicate interplay is granted by several mediators, including hormones (catecholamines, glucocorticoids) and neuronal signals, primarily through the hypothalamic–pituitary–adrenal (HPA) axis and the autonomic nervous system [13]. However, these “peripheral clocks” are also self-sustained and directly influenced by external stimuli, such as temperature changes [14]. Peripheral oscillators are influenced not only by internal stimuli, such as body temperature, but also by external stimuli. In mice, hepatocyte-intrinsic BMAL1 and hepatic farnesoid X receptor (FXR) signaling integrate nutritional cues to regulate survival in response to innate immune stimuli. [15].

It is known that many pathologic events, such as myocardial infarction, stroke, and aortic acute diseases [16,17,18,19,20,21], occur at specific times of day, indicating that circadian processes contribute to these diseases [5]. On the one hand, central circadian entrainment of peripheral clocks leads to robust daily rhythms across many physiological systems and complements homeostatic responses [5]. On the other hand, rhythm disruption due to circadian misalignment, e.g., shift work, jet lag, and even the daylight saving time (DST) shift, may contribute to several human diseases [5,22,23]. Circadian rhythm perturbations have also been observed in autoimmune diseases, such as rheumatoid arthritis (RA) and multiple sclerosis (MS) [24].

It has been reported that daily circadian rhythms can play an important role in MS. The efficiency of immune responses changes throughout the day, including immune responses directed against myelin proteins. Moreover, disruption of sleep can worsen the effects of MS, suggesting that misalignment of the circadian oscillation of biological parameters could also impact psychosocial outcomes in MS patients. Alterations in the circadian activity of cortisol and melatonin may potentially have a similar effect on MS patients by exacerbating mood-related symptoms within the context of the disease [25].

Specifically, autoimmune and inflammatory processes can directly affect clock gene expression, potentially leading to a vicious cycle of enhanced inflammation [26]. Under-standing the circadian regulation of biological processes in health and disease could in-form therapeutic strategies [27], and timed delivery of pharmaceutical compounds is gaining popularity to optimize the effectiveness of molecules already in use [28,29,30].

Therefore, the aim of the present article is to summarize the current knowledge of circadian biology in inflammatory joint diseases and discuss the potential application of chronotherapy in this evolving field.

## 2. Chronobiology and Chronotherapy in RA

### 2.1. Chronotherapy: General Principles

In recent years, a growing body of evidence has shown that molecular clocks orchestrate 24-h circadian rhythms in many vital cardiometabolic, endocrine, immunologic, and behavioral functions. Moreover, we now know that many diseases do not occur randomly throughout the 24 h period, and the incidence of heart attacks as well as symptoms in asthma, allergic rhinitis, cancer, arthritis, depression and suicidal intent, peptic ulcers and pain all show time-of-day variations [31]. However, in everyday clinical practice, most physicians administer drug treatments as needed, without considering the time of day, which, in turn, could add a potentially important dimension to medicine. Circadian medicine aims to incorporate knowledge of 24 h biological rhythms to enhance diagnosis and treatment by means of two broad strategies: targeting the molecular clock or exploiting its rhythmic outputs. In the former, the therapeutic target is the molecular oscillator itself; for example, light therapy, feeding, exercise, and drugs are capable of modulating the clock and influencing health. Moreover, thousands of rhythmically expressed genes metabolize, and transport or are the targets of drugs and depending on rhythms of absorption, distribution, metabolism and excretion, circadian time could influence drug efficacy and/or toxicity [32]. Here, we will especially discuss the modern insights of chronotherapeutic approaches to inflammatory joint diseases, keeping in mind that most evidence has been accumulated for RA, one of the most frequently diagnosed rheumatic diseases, affecting approximately 1% of the population worldwide.

### 2.2. Chronobiological Aspects of RA Pathophysiology

RA is one of the most prevalent inflammatory joint diseases and is characterized mainly by pain and swelling in the small synovial joints of the hands and feet; less frequently, patients experience extra-articular manifestations, such as rheumatoid nodules, interstitial lung disease, vasculitis, and other systemic comorbidities [33]. Proinflammatory cytokines, such as tumor necrosis factor (TNF), interleukin 1 beta (IL-1β) and interleukin 6 (IL-6), are key players in the development and progression of the disease. In particular, IL-6 represents the archetypal member of a family of cytokines involved in regulating cell survival/apoptosis, differentiation, and proliferation [34]. IL-6 is produced by a wide range of cells, including T and B cells, monocytes, mast cells, fibroblasts, osteoblasts, keratinocytes, endothelial cells, and some tumor cells [35,36]. Several studies have confirmed the crucial role of IL-6 in the development of experimental arthritis, such as murine collagen-induced arthritis (CIA) [37] and antigen-induced arthritis (AIA) [38].

Cytokines are essential for immune responses and self-defense against infection. However, once an excessive amount of cytokines is produced, serious damage, such as sepsis, autoimmunity, and allergies, may occur. Therefore, the expression of cytokines has to be tightly regulated to maintain homeostasis of the immune system via regulation at multiple levels, and the circadian clock is an important regulator of cytokines. In fact, disruption of the circadian clock appears to aggregate chronic inflammatory diseases such as RA via aberrant expression of inflammatory cytokines [39]. Moreover, disruption of the peripheral chondrocyte clock represents a recent aspect of the catabolic responses of cartilage to proinflammatory cytokines, providing an additional mechanism by which chronic joint inflammation may contribute to osteoarthritis. In fact, experimental studies on young healthy mouse cartilage showed that IL-1β (but not TNFα) abolishes circadian rhythms in Cry1-luc and PER2::LUC gene expression [40].

The initial event in the natural history of RA is the breach of tolerance. Autoantibodies are produced before the onset of articular disease, as emphasized by the evidence that rheumatoid factor (RF) and/or anti-citrullinated protein antibody (ACPA) positivity anticipates the outbreak of clinically defined arthritis [41,42]. The stimulation of autoantibody production [43] was one of the first identified immunological effects of IL-6. In support of this function, in RA patients, synovial levels of IL-6 have been shown to correlate with local levels of immunoglobulin (Ig) and, specifically, with IgM-RF [44,45,46]. Moreover, in first-degree healthy relatives of patients with RA, RF and ACPA positivity correlates with the levels of IL-6 and IL-9 [47]. Interestingly, IL-6 is also a well-recognized key player in the development of synovial ectopic lymphoid structures (ELS), which occur in approximately 40% of patients, and is associated with the local production of autoantibodies and a more severe disease phenotype [48]. In the presence of transforming growth factor-β (TGF-β), IL-6 drives the differentiation of T helper 17 (Th17) cells from naïve T cells and at the same time inhibits the generation of regulatory T (T-reg) cells [49], thus favoring the disequilibrium between pathogenic Th17 cells and the tolerogenic T-reg population [50].

In humans, the production and secretion of inflammatory cytokines show a dependence on the day–night cycle, with significant changes over 24 h [42,51]. As an example, IL-6, IFN-γ and IL-12 reach their zenith in the early morning (approximately 03:00 a.m.), and TNF-α, IL-1 and IL-10 showed higher levels in the evening (at approximately 09:00 p.m.) [51].

Substantial variability during the night has also been demonstrated for the release of IL-6 in RA patients, whose circulating levels are increased in the early morning and decline significantly from early in the afternoon to late in the evening [52]. The peak of IL-6 occurs at approximately 03:00 a.m., reaching concentrations 10 times higher than normal; this peak seems to correlate, at least in part, with the severity of morning stiffness [53]. The early morning peak of IL-6 exerts a chronic stimulus that, in turn, produces persistent stimulation of the HPA axis, with progressive insufficient production of endogenous corticotropin-releasing hormone (CRH), adrenocorticotropic hormone (ACTH), and cortisol [54], the most important endogenous anti-inflammatory mediator. In normal individuals, cortisol levels are lower at night, with a nadir between 10:00 p.m. and 02:00 a.m., and are higher in the morning (peaking at approximately 06:00 to 08:00 a.m.), following a circadian oscillation [55,56]. In RA patients, the cortisol peak was reached earlier (between 11.00 p.m. and 02.00 a.m.), at levels insufficient to compensate for the increased release of proinflammatory cytokines, realizing “relative adrenal insufficiency” [56]. Another consequence of proinflammatory cytokine release is the upregulation of hepatic 11β-hydroxysteroid dehydrogenase 1 (11β-HSD1). This enzyme converts peripheral inactive cortisone into cortisol, therefore exerting negative feedback on hypothalamic ACTH release and consequently on adrenal cortisol production [57]. Unfortunately, its potential beneficial action is balanced by the action of 11β-hydroxysteroid dehydrogenase 2 (11β-HSD2), which is highly expressed by macrophages and active T lymphocytes in inflamed tissue and is responsible for the degradation of bioactive cortisol to biologically inactive cortisone, with the results of local downregulation at inflamed sites [58,59].

Along with the circadian rhythm of cytokine secretion, disturbances in timekeeping mechanisms have been proposed in RA [60], as suggested by the abnormal expression and the lack of the antiphasic relationship of several clock genes, such as BMAL1, BMAL2, CLOCK, DEC2, NPAS2, and Cry1 [61]. For example, the upregulation of DEC2 via TNF-α, followed by an increased expression of IL-1β in the synovial membranes, enhances the proinflammatory and destructive effects of TNF-α on the RA joint [61].

To explain this phenomenon, a contribution of the pineal hormone melatonin has been postulated [62]. Patients with RA display higher baseline levels and an altered temporal profile of melatonin [63]. Indeed, melatonin levels increase progressively from evening to the early morning hours and reach a peak at midnight in patients with RA, two hours earlier than healthy controls, with a longer duration in the early morning [64]. Melatonin exerts a variety of effects on CD4+ Th1, Th17, and T-reg subsets [65]. Although some anti-inflammatory and antioxidant effects have been proposed [66], melatonin could also exert arthritogenic activity [62]. In vivo and in vitro studies demonstrated that melatonin stimulates the production of T helper type and other inflammatory cytokines (IL-1, IL-2, IL-4, IL-5, IL-6, IL-12, TNF, granulocyte-macrophage colony-stimulating factor (GM-CSF), TGF-ß and interferon (IFN)-γ) and enhances both cell-mediated and humoral responses [63,67]. The mechanisms behind these effects are incompletely understood, but it is known that melatonin attenuates Cry1 gene expression, resulting in the upregulation of cAMP production and the activation of protein kinase A (PKA) and nuclear factor kappa B (NF-kB), both crucial for the development of arthritis in animal models [59,68].

Furthermore, high levels of melatonin can affect clock disturbance by modulating ROR activation. In fact, ROR acts as a major negative regulator of inflammation and plays a central role in both BMAL and melatonin actions since ROR inhibition by REV-ERB may contribute to BMAL suppression and exacerbate RA [51,60,69]. Some studies have demonstrated that melatonin could have a beneficial anti-inflammatory effect on RA through the inhibition of RA synovial fibroblast proliferation [61], TNF and IL-1β expression [70,71] and the regulation of microRNA expression (e.g., miR-590-3p) [72].

### 2.3. Glucocorticoids and Chronotherapy of RA

Living organisms use several mechanisms to maintain biological homeostasis during environmental or physiological challenges against internal or external stress, such as the hypothalamic–pituitary–adrenal (HPA) axis, the sympathetic adrenomedullary system, and the cardiovascular, metabolic, and immune systems. The main hypothalamus–brainstem system includes corticotropin-releasing hormone (CRH) and arginine–vasopressin neurons and the locus coeruleus–norepinephrine systems [3]. The HPA axis, activated by CRH from the paraventricular nucleus of the hypothalamus via the release of corticotropin from the pituitary, stimulates the production of glucocorticoids (mainly cortisol) from the adrenal cortex. Cortisol controls a variety of physiological processes, i.e., metabolism, immune response, cardiovascular activity, and brain function. In addition to the stress stimuli response, cortisol is characterized by a robust daily rhythm, with higher circulating levels during active periods [73]. Cortisol rhythm depends on the rhythmic re-lease of ACTH, and the daily rhythm in nonstress levels of plasma cortisol usually dis-plays a 5- to 10-fold higher amplitude from the trough to peak levels in rodents [74].

According to the European League Against Rheumatism (EULAR) recommendation, disease-modifying anti-rheumatic drugs (DMARDs) are considered the gold standard treatment for RA [33]. Each DMARD has a unique mechanism of action, ultimately interfering with critical pathways in the inflammatory cascade [75]. However, glucocorticoids (GCs), usually administered at breakfast time, offer rapid symptoms and a potential dis-ease-modifying effect [76], as long-term, low-dose treatment has been shown to slow radiographic progression when given to patients with early RA, hence satisfying the conventional definition of DMARD [77]. The evidence of reduced endogenous GC production represents the theoretical basis for a chronotherapeutic approach in which “relative ad-renal insufficiency” in RA may be addressed by administering GCs as a replacement therapy at the specific time point where elevated GC levels are required to control the night peak in proinflammatory cytokines [55]. A pioneering clinical trial by Arvison et al. [78] first showed that the administration of low-dose corticosteroids during the night (2:00 a.m.) led to more pronounced and significant reductions in joint stiffness, pain, and morning serum IL-6 than traditional administration at 7:30 a.m. However, the need to break the night’s rest makes this approach difficult to apply in clinical practice [79].

In an attempt to solve this problem, a novel formulation of modified-release (MR) prednisone tablets has been introduced to counteract HPA axis insufficiency without waking the patient [80]. The MR formulation does not differ from immediate-release (IR) prednisone in terms of pharmacokinetics but only in the delayed release of the molecule after oral administration: if administered at approximately 10:00 p.m., the MR tablet releases prednisone 4 h after ingestion, intercepting the cytokine peak without the discomfort of interrupting sleep [80]. The efficacy of MR prednisone was investigated in two multicenter randomized controlled trials (RCTs) named CAPRA (Circadian Administration of Prednisone in RA).

CAPRA-1 aimed to investigate the efficacy and safety of MR prednisone compared to immediate (IR) release prednisone [81], while CAPRA-2 focused on MR prednisone as an additional GC therapy to an existing regimen including DMARDs [82]. CAPRA-1 included 288 patients with an established diagnosis of RA who were already being treated with IR prednisone and DMARDs and were randomly assigned 1:1 to take MR prednisone or IR prednisone at the same dose. After 12 weeks, the mean change in morning stiffness duration from baseline was significantly higher in the MR prednisone group than in the IR prednisone group (−22.7% vs. −0.4%, respectively, *p* = 0.045; mean absolute reduction: 44 vs. 23 min). The only other significant difference observed between treatments was the relative change in IL-6, which decreased by 28.6% upon MR prednisone, while it remained unchanged in the IR prednisone group (at week 12: *p* = 0.032), suggesting an augmented anti-inflammatory activity of the new formulation [81]. Regarding safety, MR and IR were comparable, with no difference in their rates of adverse events (AEs) (41% each). At the end of the 12-week double-blind phase, the trial was followed by a 9-month open-label extension (OLE) to evaluate the long-term efficacy and safety [83]. A total of 249 patients entered the second phase of therapy with MR prednisone: 129 continued treatments with the experimental formulation (MR/MR), and 120 switched to MR prednisone from previous IR prednisone (IR/MR) if no significant improvement had been attained. The results of the long-term OLE of CAPRA-1 were consistent with previous findings, showing that the morning stiffness reduction levels after 12 months were 45% in the IR/MR group and 55% in the MR/MR group [83].

In the following CAPRA-2 trial, 350 patients with active RA on DMARD therapy, no IR prednisone medication 6 weeks prior to screening, and a morning stiffness duration of at least 45 min were enrolled [82]. Patients were blindly randomized 2:1 to receive either 5 mg MR prednisone (*n* = 231) or placebo (*n* = 119) for 12 weeks. MR prednisone plus DMARD treatment produced higher response rates for the American College of Rheumatology improvement response criteria (48% vs. 29% for ACR20, *p* < 0.001; and 22% vs. 10%, for ACR50, *p* < 0.006) and a greater median relative reduction from baseline in morning stiffness (55% vs. 35%, *p* < 0.002) at week 12 than placebo plus DMARD treatment. Moreover, significantly greater reductions in the severity of RA (Disease Activity Score 28) (*p* < 0.001) and fatigue (Functional Assessment of Chronic Illness Therapy-Fatigue score) (*p* = 0.003) as well as a greater improvement in physical function (36-item Short Form Health Survey score) (*p* < 0.001) were seen at week 12 for MR prednisone versus placebo. Additionally, the incidence of adverse events was similar for MR prednisone (43%) and placebo (49%) [82].

Overall, low-dose MR prednisone seems to be able to improve the disease course, reduce morning stiffness and morning serum levels of IL-6, and induce a significantly higher percentage of improvement of the American College of Rheumatology response criteria ACR20 and ACR50 in combination with DMARDs compared to DMARD monotherapy [84]. One concern regarding the use of GCs is that, although powerful and widely used [85], they are frequently associated with multiple adverse effects (AEs), including bone loss, diabetes, and suppression of the HPA axis, which leads to adrenocortical hypotrophy and iatrogenic adrenal insufficiency [86]. No clinically evident AEs indicating aggravated HPA axis suppression were observed in the CAPRA trials [81,82], but to examine possible AEs of MR prednisone on the HPA axis, a small sub-study within CAPRA-1 was undertaken [80,87]. Corticotropin-releasing hormone (CRH) tests were performed on 28 patients at three timepoints: at baseline on pre-study immediate-release (IR) prednisone, after the 3-month double-blind phase on either IR prednisone or MR prednisone, and after the 9-month open-label extension on MR prednisone. Changes in cortisol were assessed and compared to individual patient efficacy and safety data. The authors found no difference in the mean increase in cortisol plasma concentrations after CRH injection at baseline compared to the end of the sub-study (5.5 ± 4.37 vs. 5.3 ± 4.07 μg/dL), and no new HPA axis suppression occurred, concluding that treatment with nighttime release prednisone did not change adrenocortical function over 12 months [87].

### 2.4. Other Chronotherapeutic Approaches in RA: Clinical and Preclinical Evidence

Methotrexate (MTX) is the anchor conventional DMARD used as a first-line treatment for RA. It stimulates adenosine release from fibroblasts, reduces neutrophil adhesion, inhibits leukotriene B4 synthesis by neutrophils, inhibits local IL-1 production, reduces the levels of IL-6 and IL-8, suppresses cell-mediated immunity, and inhibits synovial collagenase gene expression [65]. Since the circadian activation of cells involved in RA pathogenesis is the preferential target for conventional and biologic DMARDs, in this case, the administration of these drugs may be manipulated to intercept those rhythms. In a first study on an animal model of arthritis, To et al. [88] demonstrated that administering MTX at a specific time, in accordance with the 24 h rhythm of TNF release, leads to a decreased inflammatory response. More recently, in collagen-induced arthritis (CIA) rats [89], the therapeutic score was improved by administering MTX once daily, according to the circadian rhythm of IL-6. Notably, another study [90] suggested that MTX can influence the circadian environment in synovial fibroblasts by affecting the circadian transcription factor proline and acidic amino acid-rich basic leucine zipper (PAR bZIP) and the clock gene PERIOD 2 (Per2), resulting in the induction of apoptosis. In 2009, To et al. [88] investigated the potential usefulness of MTX chronotherapy in clinical practice [91]. Patients on MTX were switched from a three times/week scheme to a chronotherapy scheme, in which the dose and number of doses/wk were not changed, but MTX was administered once a day at bedtime. They found that MTX chronotherapy was associated with a significant improvement in the Disease Activity Score, including 28 joints (DAS28), at all follow-up visits, with 23.5% of patients achieving remission after three months. Moreover, the functional capacity of RA patients, as indicated by the health assessment questionnaire (HAQ), was markedly improved by chronotherapy, without additional adverse events [91].

Baricitinib is a novel Janus kinase (JAK) inhibitor approved for the treatment of moderate to severe active RA [92]. In a recent study, Yaekura et al. [93] investigated the potential benefits of baricitinib chronotherapy in CIA mice. Experimental animals were administered a dose of 3 mg/kg baricitinib once a day at zeitgeber time (ZT) 0 or ZT12, the times of lights-on and lights-off, for 21 days. Arthritis scores, histopathology, and bone destruction markers were significantly improved in the sera of mice treated at ZT0. Preclinical evidence for chronotherapy according to the results obtained in animal models of arthritis is reported in Table 1.

## 3. Chronobiology and Chronotherapy of Other Inflammatory Joint Diseases

### 3.1. Polymyalgia Rheumatica

Polymyalgia rheumatica (PMR) is the most common inflammatory rheumatic disease of the elderly. The main symptoms of PMR are pain and stiffness in the shoulders and hip girdles [96], which are typically more prominent during the early morning [97]. Low-dose GCs are the cornerstone of the treatment of PMR [98], even though in refractory cases, other medications, such as MTX, can be used as steroid-sparing agents.

Galbo et al. [99] investigated the 24 h time course of clinical symptoms in PMR and related them to the diurnal variation in the concentrations of melatonin, inflammatory cytokines, and cortisol. Ten glucocorticoid-naïve patients newly diagnosed with PMR and seven non-PMR control subjects were studied for 24 h before treatment and after 14 days of treatment with 20 mg/day prednisolone. The results revealed that PMR symptoms followed a diurnal variation, with pain and stiffness peaking in the early morning, showing a plateau between 04:00 and 08:00 and then declining to a nadir at 16:00. Plasma concentrations of IL-6, IL-8, TNF, IL-1β, and IL-4 varied with time in both groups and peaked between 04:00 and 08:00. Additionally, melatonin and cortisol were consistently higher in these patients and changed with time, peaking at approximately 02:00 and 08:00, respectively. In a prospective observational study, Benucci et al. [100] evaluated changes in inflammatory markers and their correlation with IR or MR 6-methylprednisolone (6-MP) in patients with early PMR. The results showed significant differences between baseline and end-of-treatment values in serum levels of IL-6 and CRP in patients treated with the MR regimen and in serum cortisol levels in the patients treated with 6-MP [86]. In addition, during the first month of treatment, there was a significant decrease in IL-6 levels: 76.7% of the patients treated with MR-P had IL-6 levels at or below the upper normal limit, whereas 52.6% of those treated with 6-MP had normal IL6 levels [100].

### 3.2. Gout

Gout is a chronic disease caused by monosodium urate (MSU) crystal deposition [101]. Gout typically presents as recurrent episodes of acute, self-limiting inflammatory mono-arthritis affecting the joints of the lower limbs [101]. Several factors associated with hyperuricaemia, such as medical conditions, obesity, lifestyle, and medications, are associated with the risk of developing gout [102]. Choi et al. [103] evaluated the hypothesis that gout attacks occur more frequently at night, underscoring how an accurate understanding of the circadian variation of gout could have practical implications for the effective timing of antigout prophylactic measures. They conducted a case-crossover study in which 724 gout patients were prospectively recruited and followed up via the internet for 1 year. Study participants were asked about the following information concerning their gout attacks: the date and hour of attack onset, symptoms and signs, medication used, and purported risk factors during the 24 and 48 h periods prior to the gout attack. Gout patients experienced a total of 1433 attacks in 1 year, and the results showed that the risk of gout flares in the 8 h overnight time block (12:00 a.m. to 7:59 a.m.) was 2.36 times higher than in the daytime (8:00 a.m. to 3:59 p.m.) (OR 2.36 (95% CI 2.05–2.73)). The corresponding OR in the evening (4:00 p.m. to 11:59 p.m.) was 1.26 (95% CI 1.07–1.48). The exact mechanisms have not been elucidated, but a lower body temperature, relative nocturnal dehydration, or nocturnal dip in cortisol levels may lead to an increased risk of gout attacks at night, as well as sleep apnea [104,105]. Aiming to clarify the latter relationship, a recent meta-analysis confirmed the association between obstructive sleep apnea syndrome and hyperuricaemia [106]. The application of drug prescriptions according to the principles of chronobiology underlines the recognition of the importance of chronobiologic patterns in the immune response. Immunomodulatory medications could be ad-ministered following target biological factors that are known to have circadian rhythms, and such a type of attitude emphasizes the role of chronotherapeutic techniques.

## 4. Conclusions

Although much has been learned about circadian clocks and rhythms over the past few decades and despite the milestone recognition of the field of circadian biology with the awarding of a Nobel Prize in 2017, translation to clinical practice has proceeded very slowly [107]. In recent years, also following earlier suggestions regarding the cardiovascular system [108], several studies have highlighted the advantage of administrating drugs readily used in clinical practice at a specific time to improve the efficacy and symptoms of RA [109]. In fact, on the one hand, we know that the diurnal variation in symptoms coincides with, and may be partly mediated by, a circadian variation in plasma concentrations of proinflammatory cytokines and hormones. On the other hand, this allowed the development of chronotemporized drugs, whose formulation may give the opportunity to treat patients in accordance with their biological rhythm, potentially minimizing the side effects. Thus, quite similar to sex, accounting for time of day as a biological variable should be incorporated into the design of research studies, which should include data collection and analysis of results, as well as reporting of findings, and gold-standard double-blind clinical studies should be conducted to determine the best time of day for the optimal effectiveness of medications [107]. Circadian precision medicine aiming to de-liver treatment in harmony with target physiology could improve the efficacy and safety of therapy [33].

## Figures and Tables

**Table 1:** Preclinical evidence for chronotherapy according to the results obtained in animal models of arthritis.

	Author, Year	Animal Model	Main Findings
MTX	To, 2009 [88]	CIA	Arthritis score was significantly lower in CIA mice/rats treated with a 22-HALO MTX regimen when compared to a 10-HALO group (*p* < 0.01).
	To, 2011 [91]	MRL/lpr mice	Plasma SAA levels were significantly lower in MRL/lpr mice treated with an 18-HALO MTX regimen when compared to a 6-HALO group (*p* < 0.01).
	Wang, 2018 [89]	CIA	Arthritis severity, CRP, TNF, and IL-6 were significantly lower in CIA rats treated to an 18-HALO MTX regimen when compared to a 6-HALO group (*p* < 0.01).
Baricitinib	Yaekura, 2020 [93]	CIA	Arthritis score (*p* < 0.01), TNF (*p* < 0.01) and IL-6 (*p* < 0.01) were significantly lower in CIA mice treated with BARI administered at ZT0 when compared to BARI administered at ZT12.
Tacrolimus	Obayashi, 2011 [94]	CIA, MRL/lpr	Arthritis score (*p* < 0.01) and leukocyte count (*p* < 0.01) were significantly lower in CIA and MRL/lpr mice treated with a 2-HALO TAC regimen when compared to a 14-HALO group.
Mizoribine	Kanasaki, 2012 [95]	CIA	Arthritis score (*p* < 0.01) and leukocyte count (*p* < 0.01) were significantly lower in CIA mice treated with a 22-HALO MZR regimen when compared to a 10-HALO group.

Legend: BARI, baricitinib; CIA, collagen-induced arthritis; CRP, C-reactive protein; HALO, hours after the light was turned on; IL-6, interleukin 6; MRL/lpr, homozygous for the lymphoprolifera-tion spontaneous mutation (Faslpr); MTX, methotrexate; MZR, mizoribine; SAA, serum amyloid A; TAC, tacrolimus; TNF, tumor necrosis factor; ZT, zeitgeber time.
